# Essential Oils of Plants as Biocides against Microorganisms Isolated from Cuban and Argentine Documentary Heritage

**DOI:** 10.5402/2012/826786

**Published:** 2012-10-14

**Authors:** Sofía Borrego, Oderlaise Valdés, Isbel Vivar, Paola Lavin, Patricia Guiamet, Patricia Battistoni, Sandra Gómez de Saravia, Pedro Borges

**Affiliations:** ^1^Laboratory of Preventive Conservation, National Archive of the Republic of Cuba, Compostela 906, Esquina a San Isidro, Havana, P.O. Box 10100, Old Havana, Cuba; ^2^Departamento de Química, Instituto de Investigaciones Fisicoquímicas Teóricas y Aplicadas (INIFTA), Facultad de Ciencias Exactas, Universidad Nacional de La Plata (UNLP), La Plata, Argentina; ^3^CONICET, La Plata, Argentina; ^4^Facultad de Ciencias Veterinarias, UNLP, La Plata, Argentina; ^5^Facultad de Ciencias Naturales y Museo, UNLP, La Plata, Argentina; ^6^CICBA, La Plata, Argentina; ^7^Food Industry Research Institute, Havana, Cuba

## Abstract

Natural products obtained from plants with biocidal activity represent an alternative and useful source in the control of biodeterioration of documentary heritage, without negative environmental and human impacts. In this work, we studied the antimicrobial activity of seven essential oils against microorganisms associated with the biodeterioration of documentary heritage. The essential oils were obtained by steam distillation. The antimicrobial activity was analyzed using the agar diffusion method against 4 strains of fungi and 6 bacterial strains isolated from repositories air and documents of the National Archive of the Republic of Cuba and the Historical Archive of the Museum of La Plata, Argentina. Anise and garlic oils showed the best antifungal activity at all concentrations studied, while oregano oil not only was effective against fungi tested but also prevented sporulation of them all. Orange sweet and laurel oils were ineffective against fungi. Clove, garlic, and oregano oils showed the highest antibacterial activity at 25% against *Enterobacter agglomerans* and *Streptomyces* sp., while only clove and oregano oils were effective against *Bacillus* sp. at all concentrations studied. This study has an important implication for the possible use of the natural products from plants in the control of biodeterioration of documentary heritage.

## 1. Introduction

Among the daily plagues threatening library and archival collections, the molds are certainly one of the most significant [1, 2]. Mold is harmful to paper-based documents. Active or dormant spores are present everywhere, in the air and on objects. Development of chemical biocides can be avoided by controlling the environment. Controlling the temperature and relative humidity and keeping the collections clean and free of dust and dirt are among the possible measures. Unfortunately, these preventive measures are not always correctly followed. Although the best approach to conservation is prevention, when confronted with an accident or disaster for water, fungicidal treatment must be used to disinfect the contaminated objects or environment. In the past, thymol vapour was used extensively by many conservators using a homemade chamber named “thymol cabinets.” This compound is no longer used because of its health hazard and deleterious effects on the objects. Many years ago, the European conservators had been using fumigation by ethylene oxide because of its absence of effect on cellulosic material [3]. This type of treatment needs special vacuum chambers and must be executed by specialists because this compound is very dangerous to environment and human health.

The essential oils in the vapour phase or blended with some solvent have the potential to be used as a fumigant and can be an interesting alternative, since antiquity, volatile oils from herbs, species, and plants have been recognized as having biological activities. In recent years, there has been renewed interest by scientists in the use of these natural substances for their antibacterial and antifungal properties. However, among several studies reported, only a few mention their eventual use in the field of conservation of cultural properties [4–6].

The aim of this work was to evaluate the antimicrobial activity of seven essential oils from plants against microorganisms associated with the biodeterioration of documentary heritage.

## 2. Materials and Methods

### 2.1. Obtaining Essential Oils

The oils were provided by the Food Industry Research Institute, Havana, Cuba. All were extracted by hydrodistillation using Clevenger type apparatus for 3 h. Seven essential oils of the plants *Pimpinella anisum* L. (anised), *Syzygium aromaticum* L. (clove), *Cuminum cyminum* L. (cumin), *Allium sativum* L. (garlic), *Laurus nobilis* L. (laurel), *Citrus sinensis* (L.) Osbeck (orange sweet), and *Origanum vulgare* L. (oregano) were analyzed.

### 2.2. Analysis of Essential Oils by Gas Chromatography-Mass Spectrometry (GC-MS)

For GC/MS analysis, an HP 6890 Series II equipped with a mass-selective detector HP-5973N and an HP-5MS-fused silica column (25 m × 0.25 mm × 0.25 *μ*m film thickness) were employed. The column temperature was programmed as follows: 70°C holds 2 min to 230°C at 4°C/min and then holds 10 min. Helium carrier gas was used at a flow rate of 1 mL/min. The injector was maintained at 230°C. Sample injection volume was 0.3 *μ*L with a split ratio of 1 : 10. Mass spectra were recorded in the electron-impact (EI) mode at 70 eV by 1.8 scans/s; the mass range used was m/z 35–400; ion source and connecting parts temperature were 230°C. Linear retention indices (RIs) were calculated using n-paraffin standards.

Compounds were preliminarily identified by comparison of mass spectra with those of reference standards (FLAVORLIB library) or those in NIST, NBS/Wiley, and mass spectra from the literature, and then the identities of most were confirmed by comparison of their linear retention indices with those of reference standards or with published data [7, 8].

Quantitative analysis was made by the normalization method from the electronic integration of the TIC peak areas without the use of correction factors.

### 2.3. Microorganisms and Growth Conditions

The experiments were carried out with fungal and bacterial strains isolated from different documentary supports and indoor environments of repositories of the National Archive of Republic of Cuba and Historical Archive of the Museum of La Plata, Argentina [9–11]. The bacterial strains used were *Bacillus polymyxa, Bacillus cereus, Bacillus thuringiensis, Bacillus* sp., *Enterobacter agglomerans, *and *Streptomyces* sp. The fungal strains were *Aspergillus niger, Aspergillus clavatus, Penicillium* sp., and *Fusarium* sp. They were maintained on malt extract agar (MEA) slants, and the bacterial species had been maintained on nutrient agar (NA) slants. 

### 2.4. Antimicrobial Activity Assay

The antimicrobial activity of the essential oils was evaluated by hole-plate diffusion methods [12, 13]. Suspensions of the bacterial strains used were adjusted to tube 1 of the McFarland scale, and the Petri dishes with NA were inoculated with a final concentration of 10^5^ CFU/mL [14]. For the fungi, suspensions of conidia were adjusted using a Neubauer's chamber to 10^6^ conidia/mL [15], and the Petri dishes with MEA were inoculates with a final concentration of 10^4^ conidia/mL [16]. Six holes of 5 mm of diameter (*d*) were made halfway in each Petri dish of 110 mm, and 10 *μ*L of each essential oil pure or dissolved in ethanol (70%) at different concentrations was added. Additional holes with ethanol at 70%, gentamycin sulphate at 40 mg/mL (Medical-Pharmaceutical Industry, Cuba), and miconazole at 10 mg/mL (Medical-Pharmaceutical Industry, Cuba) were used as controls. Each experiment was done in triplicate.

For bacteria, the dishes were incubated at 30°C for 24 h to 72 h, and the dishes with fungal strains were incubated at 28°C during 5 days. Having finished the time of incubation, the diameter of the inhibition zone was measured and it was not included in 5 mm of the holes. The established range to determine susceptibility to oil was evaluated according to the diameter of inhibition zone, (d) *d* ≤ 6 mm is indicative of negative activity; *d* = 7–10 mm indicates a moderate activity; *d* ≥ 11 mm indicates a positive activity [13].

## 3. Results

Table 1 shows the major components detected in the essential oils studied. Different compounds such as aldehydes (e.g., cuminaldehyde in cumin oil and citral in orange sweet oil), phenolic compounds (e.g., eugenol, carvacrol), alcohols (e.g., terpineol, linalool), and monoterpenes (e.g., anethole, eugenol) were detected.

Determination of the inhibition zones by means of the hole-plate diffusion methods by bacteria (Table 2) showed that the oils exhibit different antibacterial effects. The essential oil of clove showed positive activity (≥11 mm) against *Bacillus cereus, Bacillus thuringiensis, *and *Streptomyces* sp., while this oil exhibited a moderate activity against *Bacillus polymyxa*. The garlic oil was very effective against *Bacillus polymyxa, Enterobacter agglomerans,* and *Streptomyces* sp. at the two concentrations studied as long as this oil showed a high activity only against *Bacillus* sp. and *B. polymyxa* at 50%. Against *Bacillus cereus* and *Bacillus thuringiensis,* the garlic oil was less effective. 

On the other hand, the oregano oil at 50% (v/v) was effective against all strains analyzed and *Bacillus cereus, Bacillus thuringiensis* and *Streptomyces* sp. were very sensitive at 25% too. It is very important to point out that at 25% this oil exhibited the highest activity against *Bacillus cereus *and *Bacillus thuringiensis.* Only the orange sweet oil was effective against *Streptomyces* sp. The rest of the other essentials oils studied did not show significant inhibitory activities for the bacterial strains examined.

In relationship to the fungal strains studied (Table 3), the garlic and anise oils were the best because they exhibited the highest activities against all strains at the five concentrations studied. The clove, cumin and oregano oils exhibited a high activity until 25% for all strains; likewise, also the sporulation of all fungi tested was inhibited by clove and oregano oils. Orange sweet and Laurel oils did not show antifungal activity for none of the strains tested but only the fungi sporulation of *A. niger* and *A. clavatus* were inhibited by laurel.

**Figure 1:**
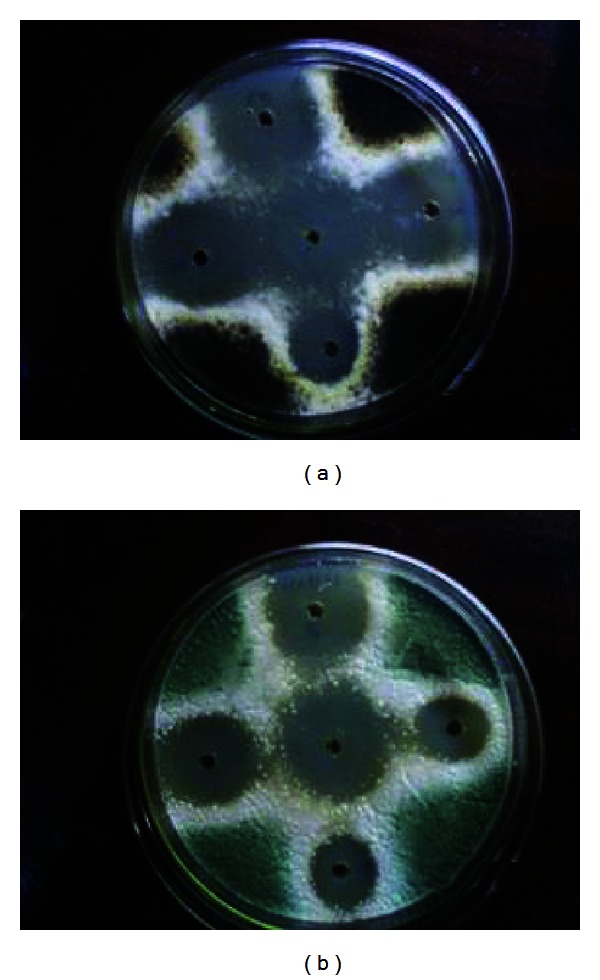
Inhibition halo of growth of *A. niger* (a) and *Penicillium* sp. (b) against oregano oil at five concentrations studied (100, 50, 25, 12.5, and 7.5%). Also, it can be observed that the inhibition of the fungal sporulation in the next area is due to the lack of growth.

Also, it was observed that clove and oregano oils also stopped the fungal sporulation in the next area to the lack of growth, while laurel oil only exhibited inhibition of the sporulation (Figure 1).

## 4. Discussion

Essential oils of many plants are known to have antimicrobial activity [16]. For this reason, some scientists of the conservation have evaluated some essential oils against microorganisms isolated from documents and environments of archives, libraries, and museums [4–6]. However, the studies that use essential oils for the conservation of the cultural heritage are still not enough.

The essential oils selected in this study have antimicrobial, antiseptic, and disinfectant actions, given by their contents in terpenes, aromatic aldehydes, terpenic aldehydes and phenolic compounds, among other components (Table 1) which are evaluated by different authors previously [17, 18].

Anise oil had no antimicrobial activity on bacteria, or the property was very low (Table 2). Similar results were obtained by Chaudhry and Tariq [19]. This behaviour may be due to a problem of oil solubility because it is known that the different susceptibility of the bacteria to the substances may be due to variations in the cell wall structure, lipid, and protein composition of the cytoplasmic membrane as well as in specific physiological processes [17]. The main compound of anise is anethole, and this compound is characterized by amphiphilic properties, which allows the interaction with the cytoplasmic membrane, membrane fluids, proteins, lipids, and other molecules vital to microbial cells [20]. Contrary to the situation in the bacteria, anise oil showed a positive activity against all fungal strains studied (Table 3). The antifungal activity of anise has been reported by different authors [21, 22]. 

Clove oil also has antibacterial and antifungal activity, but antibacterial activity obtained in our study was variable. Most Gram-positive bacteria were sensitive to this oil, and *Enterobacter agglomerans* (Gram negative) was resistant. This may be due to the characteristics of the cell wall. Instead, this oil showed a high antifungal activity at concentrations up to 25% (v/v). Similar results were reported by different authors in the conservation of documentary heritage [5, 6]. However, the inhibition obtained in this work was not as significant as that reported by Rakotonirainy and Lavédrine [6], possibly due to differences in oil quality. The antimicrobial activity of clove is attributed to eugenol (2-methoxy-4-allyl phenol). Clove bud oil contains high eugenol content (70%). It is an antimicrobial compound having wide spectra of antimicrobial effect [23].

An antibacterial negative activity was obtained with cumin oil, contrary to those reported previously by other authors [24]. Those authors reported that cumin showed antibacterial activity only against some species of bacteria, both Gram positive and Gram negative. However, the fungi analyzed were more sensitive than bacteria; opposite results were reported by Shetty et al. [25]. However, Marjanlo et al. [26] reported a high activity of this oil against the fungal species *Botrytis cinerea* and attributed this effect to the cuminaldehyde. Therefore, we can infer that the good results obtained in this study with fungi may be due to the high concentration of cuminaldehyde in the oil tested.

The antibacterial activity of garlic was very variable between Gram positive bacteria, as against *Bacillus cereus* and *Bacillus thuringiensis* they were less effective, whereas for *Bacillus* sp. and *Bacillus polymyxa* they showed positive activities. This could be due to the possible resistance that bacterial spores have [12]. However, compared to *Streptomyces* sp., the activity was positive. On the other hand, the antifungal activity was higher. Action is likely to be due to allicin and ajoene [27], which are substances that inhibit the activity of enzymes sulphydrics (choline esterase, urease, dehydrogenase triphosphate, etc.) and not sulphydrics (lactate dehydrogenase, alkaline phosphatase) of the microorganisms [28]. But these compounds were not detected, and it is possible that the antifungal activity was shown at the expense of the sulfides present in the oil.

Laurel oil showed a low antibacterial and antifungal activity contrary to the report of Alpsoy [22]. However, since the maximum concentration of these oils, the sporulation of the two strains of* Aspergillus* was inhibited (Table 3).

With the oregano oil, no significant differences were obtained between bacterial strains, Gram positive and Gram negative, analysed, but the activity was high in all cases. Reports were similar to different species of *Bacillus* [29, 30]. However, for fungi, not only it was effective for growth, but also it inhibited sporulation of all strains studied. Similar results were obtained previously [30–32]. This could be due to the presence of thymol, which can act on the bacterial membrane and fungal ultrastructure [14].

## 5. Conclusions

The essential oils were more effective against fungi analyzed than the bacteria tested, although fungi are generally more resistant to antimicrobial. Only essential oils of sweet orange and laurel had a negative activity against fungal strains. Of the seven essential oils studied, two (anise and clove) were highly effective against fungi tested.

This study has an important implication for the possible use of these vegetable biocides in the control of biodeterioration of documentary heritage after studying its effects on the chemical, molecular, structural, and aesthetic characteristics in the paper.

## Figures and Tables

**Table 1:** List of essential oils tested and the major components detected in each one.

Common name	Botanical correspondence	Percentage composition of major components
Anise seed	*Pimpinella anisum *L*. *	Anethole (80.8%), methyl chavicol (2.5%), methyl eugenol (2.4%), linalool (2.3%), and acetanisole (2.0%)

Clove	*Syzygium aromaticum *L.	Eugenol (67.0%), eugenyl acetate (18.1%), methyl *o*-hidroxibenzoate de metilo (9.0%), and anethole (5.9%)

Cumin	*Cuminum cyminum *L.	Cuminaldehyde (43.3%), cuminal (20.4%), *β*-pinene (12.8%), *α*-terpinene (4.5%), carbicol (4.4%), p-cymene (2.7%), *γ*-terpinene (2.5%), and thymol (2.3%)

Garlic	*Allium sativum *L.	Di-2-propenyl trisulfide (31.9%), methyl 2-propenyl trisulfide (21.7%), di-2-propenyl disulfide (20.7%), di-2-propenyl sulfide (7.9%), methyl 2-propenyl disulfide (5.6%), methyl 2-propenyl sulfide (5.6%), and dimethyl trisulfide (1.6%)

Laurel	*Laurus nobilis *L.	1,8-Cineole (26.7%), eugenol (18.5%), linalool (18.5%), sabinene (11.8%), methyl eugenol (6.5%), *β*-pinene (2.4%), *α*- terpineol (1.2%), and *β*-caryophyllene (1.1%)

Orange sweet	*Citrus sinensis *(L.) Osbeck	D-limonene (82.7%), *α*-pinene (2.4%), citral (1.8%), and citronellol (1.5%)

Oregano	*Origanum vulgare *L.	Thymol (38.0%), cis-*β*-terpineol (16.5%), terpinen-4-ol (10.2%), *γ*-terpinene (7.3%), *α*-terpinene (4.3%), p-cymene (3.7%), sabine (3.7%), and carvacrol (3.4%)

**Table 2:** Median values of the antibacterial activity of seven essential oils dissolved in ethanol at 70% (v/v) at two concentrations against six bacterial strains isolated from documents biofilms and indoor environments of archival repositories.

	Details of plant oils		Diameter of inhibition zone (mm) at 24 h^∗a^					
Plant species	Common name	Conc. (%)	*Bacillus* sp.	*Bacillus polymyxa *	*Bacillus cereus *	*Bacillus thuringiensis *	*Enterobacter agglomerans *	*Streptomyces* sp.
*Pimpinella anisum *	Anise	50	0	2	0	5	4	5
		25	0	5	0	7	6	7
*Syzygium aromaticum *	Clove	50	7	10	14	18	6	11
		25	7	10	18	24	6	12
*Cuminum cyminum *	Cumin	50	4	5	0	0	5	8
		25	4	4	0	0	5	4
*Allium sativum *	Garlic	50	15	14	8	8	30	30
		25	10	11	8	7	30	30
*Laurus nobilis *	Laurel	50	10	4	0	0	2	4
		25	7	4	0	0	2	2
*Citrus sinensis *	Orange sweet	50	7	6	8	8	1	12
		25	10	5	7	8	1	12
*Origanum vulgare *	Oregano	50	12	11	25	21	11	14
		25	10	10	30	30	8	11
Controls	Ethanol	70	0	0	0	0	0	0
	Gentamycin sulphate	40 mg/mL	17	20	18	21	26	32

*Streptomyces sp. was incubated at 72 h.

^a^Data are mean of three replications.

**Table 3:** Median values of the antifungal activity of seven essential oils dissolved in ethanol at 70% (v/v) against four fungal strains isolated from documents biofilms and indoor environments of archival repositories.

Essential oils		Diameter of inhibition zone (mm) at 5 days^a^			
(Common name)	Conc. (%)	*Aspergillus niger*	*Aspergillus clavatus*	*Penicillium *sp.	*Fusarium *sp*. *
Anise	100	40	40	40	40
	50.0	40	40	40	40
	25.0	40	40	40	40
	12.5	40	40	40	40
	7.5	40	40	40	40

Clove	100	20*	15*	20*	30*
	50.0	15*	13*	15*	20*
	25.0	13*	12*	13*	18*
	12.5	11*	7*	6*	15*
	7.5	8*	6*	5*	14*

Cumin	100	18	25	15	15
	50.0	15	20	13	12
	25.0	12	13	12	11
	12.5	11	7	0	0
	7.5	10	5	0	0

Garlic	100	40	40	40	40
	50.0	40	40	40	40
	25.0	40	40	40	40
	12.5	40	40	40	40
	7.5	40	40	40	40

Laurel	100	5*	8*	7	6
	50.0	0**	0**	0	0
	25.0	0**	0**	0	0
	12.5	0**	0**	0	0
	7.5	0**	0**	0	0

Orange sweet	100	5	5	6	7
	50.0	3	0	5	5
	25.0	0	0	0	0
	12.5	0	0	0	0
	7.5	0	0	0	0

Oregano	100	30*	30*	30*	30*
	50.0	20*	25*	20*	25*
	25.0	15*	15*	15*	15*
	12.5	15*	15*	15*	15*
	7.5	5*	10*	15*	15*

Ethanol	70	0	0	0	0

Miconazole	10 mg/mL	6	6	10	11

*It indicates that the oil analyzed also stop due to the fungal sporulation in the next area due to the lack of growth. **It indicates that the oil analyzed only inhibited sporulation of the fungi. ^a^Data are mean of three replications.
